# Comparison of the clinical features and long-term prognosis of gallbladder neuroendocrine carcinoma versus gallbladder adenocarcinoma: A propensity score-matched analysis

**DOI:** 10.17305/bb.2023.9582

**Published:** 2023-12-01

**Authors:** Ya-Fei Hu, Wen-Jie Ma, Hai-Jie Hu, Han-Fei Gu, Yan-Wen Jin, Fu-Yu Li

**Affiliations:** 1 Department of Biliary Surgery, West China Hospital, Sichuan University, Chengdu, China; 2 Department of Biliary Disease Research Center, West China Hospital, Sichuan University, Chengdu, China

**Keywords:** Gallbladder neuroendocrine carcinoma (GBNEC), gallbladder adenocarcinoma (GBADC), propensity score matching (PSM), overall survival (OS), prognosis

## Abstract

Gallbladder neuroendocrine carcinoma (GBNEC) is rare and characterized by a low degree of tumor differentiation. The clinical features of GBNEC versus gallbladder adenocarcinoma (GBADC) remain a subject of debate. A total of 201 GBADC and 36 GBNEC cases that underwent surgery resection between January 2010 and 2022 at the Department of Biliary Surgery, West China Hospital, Sichuan University were included. A 1:1 propensity score matching (PSM) was performed based on seven predefined variables: age, sex, the American Joint Committee on Cancer (AJCC) stage, resection status, perineural invasion (PNI), lymphovascular invasion (LVI), and degree of tumor differentiation. Compared with GBADC, GBNEC patients were younger (median age 56.0 vs 64.0 years; *P* ═ 0.001), and more patients presented with advanced stages of tumor (*P* ═ 0.003). Patients with GBNEC also had a higher rate of PNI (55.6% vs 22.4%; *P* < 0.001), and LVI (63.9% vs 45.80%; *P* ═ 0.658). Before PSM, GBNEC patients had inferior prognoses compared with GBADC patients with a shorter median overall survival (mOS) (15.02 vs 20.11 months; *P* ═ 0.0028) and a shorter median recurrence-free survival (mRFS) (10.30 vs 15.17 months; *P* ═ 0.0028). However, after PSM analyses, there were no differences in OS (mOS 18.6 vs 18.0 months; *P* ═ 0.24) or RFS (mRFS 10.98 vs 12.02 months; *P* ═ 0.39) between the GBNEC and GBADC cases. After multivariate analysis, tumor diagnosis (GBNEC vs GBADC) was not identified as an independent risk factor for shorter RFS (*P* ═ 0.506) or OS (*P* ═ 0.731). Unfavorable pathological features, including advanced AJCC tumor stages, poor differentiation, presence of LVI, and positive resection margins (all *P* < 0.05), were independent risk factors for inferior OS and RFS. GBNEC is difficult to diagnose early and has a prognosis comparable to stage-matched poorly differentiated GBADC. Tumor diagnosis (either GBADC or GBNEC) was not an independent risk factor for the patient’s OS. Unfavorable pathological features of the neoplasm are the main determinants.

## Introduction

Gallbladder neuroendocrine carcinoma (GBNEC) is extremely rare [1–3], accounting for less than 2% of all gallbladder cancer [4–6]. It is not known whether there is a significant difference between GBNEC and conventional gallbladder adenocarcinoma (GBADC). Some literature has found more aggressive tumor behavior and poorer prognosis for GBNEC patients [7–10], while other published studies reported comparable clinical features and prognoses for patients with GBNEC and GBADC [11]. The characteristics and prognosis of GBNEC have not been determined [12–14].

According to the World Health Organization (WHO) classification [1], GBNEC is a group of poorly differentiated neuroendocrine neoplasms characterized by a high proliferation index (assessed by Ki-67) [4], diffuse solid growth patterns, and frequent necrosis [15–19]. On the contrary, GBADC shows various degrees of differentiation, including well-differentiated, poorly differentiated, and undifferentiated forms [7]. We hypothesize that the published conflicting results may be related to the different degrees of differentiation between GBADC and GBNEC. In the studies by Chen et al. [8] and Yan et al. [9], both authors found a significantly longer overall survival (OS) for GBADC patients compared to those with GBNEC. In the study by Yan et al., 40% of GBADC cases were well-differentiated, and in Chen et al.’s study, 54.11% of GBADC cases were intermediate or well-differentiated. It is well known that patients with well-differentiated tumors have a better prognosis than those with poorly differentiated carcinoma [20–23]. Recently, two propensity score matching (PSM) analyses by Yan et al. [9] and Do et al. [11] were published. However, when comparing with GBNEC, these studies also failed to take the differentiation degrees of GBADC into account.

We performed a 1:1 PSM analysis using seven predefined variables: patient age, sex, perineural invasion (PNI), lymphovascular invasion (LVI), tumor differentiation degree, the American Joint Committee on Cancer (AJCC) stage, and resection status to compare differences between patients with GBADC and GBNEC. We also analyzed the prognostic factors for OS and recurrence-free survival (RFS) in patients with gallbladder cancer.

## Materials and methods

### Patients selection

The research was conducted by a referral medical center in China (Department of Hepatobiliary Surgery, West China Hospital, Sichuan University). As the study is retrospective, the need for informed consent was waived by our ethics committee. A database was collated retrospectively between January 2010 and 2022 to retrieve patients who had undergone surgical resections and had pathologically confirmed GBADC and GBNEC for the analyses. The tumor diagnostic criteria were mainly based on the WHO 2019 classification [21, 22].

**Figure 1.**
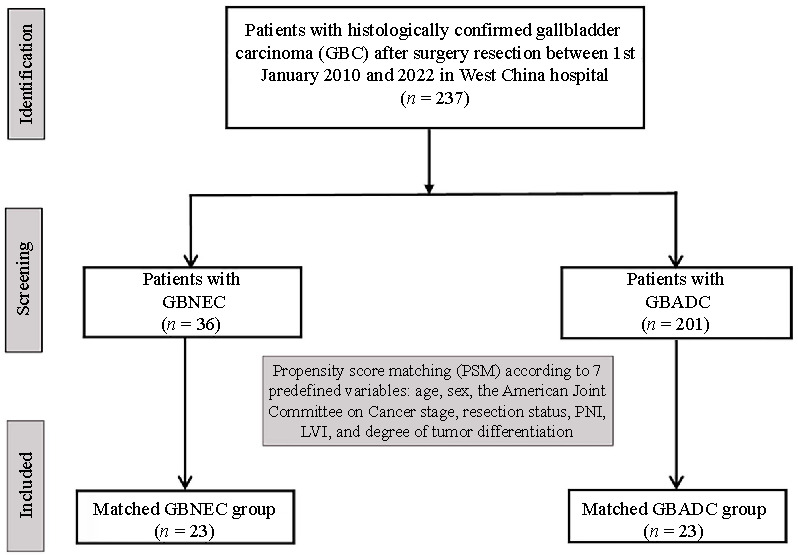
**The study flow diagram.** GBNEC: Gallbladder neuroendocrine carcinoma; GBADC: Gallbladder adenocarcinoma; PNI: Perineural invasion; LVI: Lymphovascular invasion.

The inclusion criteria were: 1) the primary tumor originated from the gallbladder; 2) patients diagnosed with GBNEC based on pathology and immunohistochemistry results, in accordance with the 2019 WHO classification; and 3) accurate and complete medical records and important clinical data for the included patients. The exclusion criteria were as follows: 1) cases with neuroendocrine carcinoma (NEC) arising from the extrahepatic bile ducts or the cystic duct, or metastasis from other organs, such as the liver or lung; 2) patients with incomplete medical records; 3) cases with <30% of the NEC component or well-differentiated neuroendocrine neoplasms with a Ki-67 <20%. The diagnosis for the included patients was independently confirmed by two gastrointestinal pathologists after surgery. Neuroendocrine markers, including synaptophysin, chromogranin [1], CD56, and Ki-67 staining, were performed on most included cases. In order to reduce the selection bias, we only included cases of GBNEC that had undergone surgical resection at our center during the same time period as the control group (GBADC). The study flow diagram is shown in Figure 1.

Clinical information collected include: age at diagnosis, sex, laboratory examination outcomes before the operation, treatment methods, and tumor stages (based on the eighth AJCC staging system for biliary tract cancers) both before and after PSM analyses. These data are presented in Table 1.

### Definitions

According to the WHO 2019 classification [1] for neuroendocrine tumors of the gastrointestinal tract, GBNEC tumors were classified as pure GBNEC or mixed adeno-neuroendocrine carcinomas (GBMANEC) based on the proportion of glandular components. If a tumor had at least 30% of both GBNEC and GBADC components, it was classified as GBMANEC. The normal range for cancer antigen 19-9 (CA 19-9) is between 0 and 37 U/mL. In our study, patients were grouped into CA 19-9 ≥ 37.0 vs < 37.0 U/mL (elevated vs normal). The normal range for carbohydrate antigen 125 (CA 125) is between 0 and 35 U/mL. In our study, patients were grouped into CA 125 ≥ 35.0 vs < 35.0 U/mL (elevated vs normal).

The duration of each patient’s follow-up was defined as the interval from the date of diagnosis to the last examination date or the date of last follow-up. OS was considered as the interval from the date of surgery to the date of death or the most recent follow-up. RFS was considered as the interval from the date of surgery to recurrence, metastasis, or last follow-up if recurrence did not occur during follow-up.

### Ethical statement

This study was conducted in accordance with the Declaration of Helsinki and in compliance with the study protocol and ethical guidelines for medical and health research involving human subjects. The study was approved by the Institutional Review Committee of West China Hospital, Sichuan University (approval code 2021-445). A summary of the study protocol is available on the website of the hospital.

### Statistical analysis

Data on the tumor parameters and patients’ demographics are expressed as mean values for parametric continuous data, and as median values for data with a non-parametric distribution. Categorical data are expressed as percentage frequencies *N* (%). The distribution of variables was analyzed using the Kolmogorov–Smirnov test.

To decrease the selection bias inevitably associated with retrospective studies, we carried out a PSM analysis between the GBNEC and GBADC groups. Based on background knowledge, variables related to gallbladder cancer patients’ prognoses were selected for the PSM analyses. We calculated the propensity score using factors that included patient age, sex, tumor differentiation degree, the AJCC stage, and resection status. Setting a caliper width of 0.25 standard deviations of the propensity score, GBNEC cases were matched to GBADC controls without replacement to the closest matched propensity score in a 1:1 ratio [24]. The statistical significance of differences in the final matched groups (GBNEC and GBADC) was assessed at the *P* < 0.05 threshold.

The chi-square test was used to compare differences between categorical variables. The unpaired student’s *t*-test was used to compare differences between continuous parametric variables. Fisher’s exact test, chi-squared test, or Mann–Whitney *U* test were used to make comparisons between the groups, as appropriate. Long-term OS and RFS were estimated using Kaplan–Meier curves, and comparisons were performed with the log-rank test. Based on background knowledge, factors related to long-term outcomes were selected for univariate and multivariable analyses. All factors that were significant in the univariate analysis were then entered into a multivariate Cox proportional hazards model.

A two-tailed *P* value < 0.05 was set for statistical significance. R software package version 3.4.2 (The R Foundation for Statistical Computing, Vienna, Austria) was used for the analyses.

## Results

A total of 201 GBADC and 36 GBNEC cases were included for analysis. In accordance with the WHO 2019 classification, eight patients diagnosed with GBMANEC were included in the GBNEC group. Table 1 summarizes the clinical and histologic features of the included GBNEC cases. Table 2 shows the characteristics of GBNEC and GBADC before and after PSM.

**Table 1:** Clinical features of included GBNEC

**Characteristics**	**GBNEC**
	***N* ═ 36**
Age, years (mean [SD])	56.00 (48.25, 61.25)
*Sex, N (%)*	
Female	26 (72.2)
Male	10 (27.8)
*Resection margins, N (%)*	
R0	27 (75.0)
R1	9 (25.0)
*Laboratory parameters (median [IQR])*	
ALT, U/L	27.00 (13.75, 66.50)
AST, U/L	23.50 (18.00, 50.25)
TB, µmol/L	11.20 (8.85, 14.17)
WBC, cells x 10^9^/L	6.48 (4.96, 7.79)
*Tumor markers (median [IQR])*	
AFP, ng/mL	3.14 (2.24, 4.73)
CA 125, U/mL	16.36 (0.00, 27.00)
CA 19-9, U/mL	14.28 (8.08, 38.53)
*Clinical symptoms, N (%)*	
*Abdominal discomfort*	
Absence	14 (38.89)
Presence	22 (61.11)
*Weight loss*	
Absence	25 (69.44)
Presence	11 (30.56)
*NEN classification, N (%)*	
NEC, small-cell type	25 (69.44)
NEC, large-cell type	3 (8.33)
GBMANEC	8 (22.23)
*Degree of differentiation, N (%)*	
Grade 3	36 (100)
*Immunohistochemical stain, N (%)*	
*CD56*	
Negative	9 (25)
Positive	27 (75)
*Synaptophysin*	
Negative	5 (13.9)
Positive	31 (86.1)
*Chromogranin*	
Negative	7 (19.4)
Positive	29 (80.6)
*Ki-67 index, median (SD)*	64.26 (17.41)
LVI, *N* (%)	23 (63.9)
PNI, *N* (%)	20 (55.6)
*TNM stages, N (%)*	
II	2 (5.6)
IIIA	6 (16.7)
IIIB	8 (22.2)
IVA	6 (16.7)
IVB	14 (38.9)

CA 19-9: Cancer antigen 19-9; NEC: Neuroendocrine carcinoma; TNM: Tumor node metastasis; GBMANEC: Gallbladder mixed adeno-neuroendocrine carcinoma; CA 125: Carbohydrate antigen 125; AFP: Alpha-fetoprotein; TB: Total bilirubin; WBC: White blood cell; PNI: Perineural invasion; LVI: Lymphovascular invasion; GBNEC: Gallbladder neuroendocrine carcinoma.

**Table 2:** Clinical characteristics of GBNEC and GBADC (before and after propensity score matching)

**Variable**	**Before propensity score matching**			**After propensity score matching**		
	**GBADC (*n* ═ 201)**	**GBNEC (*n* ═ 36)**	***P* value**	**GBADC (*n* ═ 23)**	**GBNEC (*n* ═ 23)**	***P* value**
Age, years (median [IQR])	64.00 (52.00, 70.00)	56.00 (48.25, 61.25)	**0.001**	59.00 (50.50, 64.00)	58.00 (51.50, 62.00)	0.991
*Sex, N (%)*						
Female	129 (64.2)	26 (72.2)	0.457	13 (56.5)	17 (73.9)	0.353
Male	72 (35.8)	10 (27.8)		10 (43.5)	6 (26.1)	
*TNM stages, N (%)*			**0.003**			0.823
I	17 (8.5)	0 (0.0)		0 (0.0)	0 (0.0)	
II	21 (10.4)	2 (5.6)		3 (13.0)	2 (8.7)	0.688
IIIA	33 (16.4)	6 (16.7)		4 (17.4)	3 (13.0)	
IIIB	78 (38.8)	8 (22.2)		4 (17.4)	6 (26.1)	
IVA	25 (12.4)	6 (16.7)		7 (30.4)	4 (17.4)	
IVB	27 (13.4)	14 (38.9)		5 (21.7)	8 (34.8)	
*Differentiation, N (%)*			**<0.001**			
High	31 (15.4)	0 (0.00)		–	–	
Moderate	52 (25.9)	0 (0.00)		–	–	
Poor	118 (58.7)	36 (100.00)		23 (100.0)	23 (100.0)	NA
*Resection margins, N (%)*						
R0	171 (85.1)	27 (75.0)	0.209	18 (78.3)	19 (82.6)	1
R1	30 (14.9)	9 (25.0)		5 (21.7)	4 (17.4)	
LVI, *N* (%)	92 (45.8)	23 (63.9)	0.068	15 (65.2)	16 (69.6)	1
PNI, *N* (%)	45 (22.4)	20 (55.6)	**<0.001**	12 (52.2)	12 (52.2)	1
TB, µmol/L (median [IQR])	11.90 (9.10, 16.60)	11.20 (8.85, 14.17)	0.513	10.80 (7.30, 16.25)	10.10 (8.60, 13.30)	0.758
ALT, U/L (median [IQR])	24.00 (16.00, 40.00)	27.00 (13.75, 66.50)	0.624	30.00 (22.00, 52.00)	28.00 (18.00, 74.50)	0.676
AST, U/L (median [IQR])	25.00 (20.00, 38.00)	23.50 (18.00, 50.25)	0.837	30.00 (24.00, 55.00)	24.00 (20.00, 59.00)	0.227
CA 125, U/mL (median [IQR])	21.28 (12.65, 48.83)	16.36 (0.00, 27.00)	**0.006**	1.88 (12.98, 73.35)	16.09 (0.00, 26.78)	0.031
CA 19-9, U/mL (median [IQR])	21.35 (11.83, 211.80)	14.28 (8.08, 38.53)	**0.024**	25.87 (12.88, 143.70)	14.28 (8.41, 31.99)	0.082
AFP, mg/mL (median [IQR])	2.83 (1.85, 4.32)	3.14 (2.24, 4.73)	0.202	2.85 (2.05, 3.93)	2.46 (2.01, 4.26)	0.829
WBC, cells x 10^9^/L (median [IQR])	6.13 (5.11, 7.82)	6.48 (4.96, 7.79)	0.92	5.89 (4.86, 7.52)	7.62 (5.81, 9.71)	0.222
CEA, ng/mL (median [IQR])	2.86 (1.52, 8.82)	2.58 (1.58, 9.21)	0.88	3.63 (2.16, 20.91)	3.99 (2.04, 11.32)	0.692
DBIL, µmol/L (median [IQR])	4.40 (3.20, 6.20)	2.60 (2.20, 3.60)	**<0.001**	4.20 (2.85, 5.30)	2.50 (2.15, 3.60)	0.002
Jaundice, *N* (%)	25 (12.6)	0 (0.0)	0.05	5 (21.7)	0 (0.0)	0.058
IGBC, *N* (%)	68 (33.8)	0 (0.0)	**<0.001**	6 (26.1)	0 (0.0)	0.029
Gallstone, *N* (%)	85 (42.3)	16 (44.4)	0.954	8 (34.8)	7 (30.4)	1
Adjuvant radio-chemotherapy, *N* (%)	129 (64.2)	26 (72.2)	0.457	5 (21.7)	4 (17.4)	1

Bold values indicate statistical significance. CA 19-9: Cancer antigen 19-9; CEA: Carcinoembryonic antigen; TNM: Tumor node metastasis; CA 125: Carbohydrate antigen 125; AFP: Alpha-fetoprotein; TB: Total bilirubin; WBC: White blood cell; DBIL: Direct bilirubin; PNI: Perineural invasion; IGBC: Incidental gallbladder cancer; LVI: Lymphovascular invasion; GBNEC: Gallbladder neuroendocrine carcinoma; GBADC: Gallbladder adenocarcinoma.

### Clinical features of GBNEC and GBADC before and after propensity score matching

#### Before propensity score matching

Most of the patients’ baseline characteristics and tumor oncological features were comparable (Table 2). The main differences between the unmatched cohorts were that GBNEC patients were younger (median 56.0 years, range 48.25–61.25 years) than those with GBADC (median 64.0 years, range 52.0–70.0 years) (*P* ═ 0.001) and were associated with significantly lower direct bilirubin levels (median 2.6 vs 4.4 µmol/L, *P* < 0.001) and lower levels of serum tumor markers CA 125 (median 16.36 vs 21.28 U/mL, *P* ═ 0.006) (normal value < 35 U/mL) and CA 19-9 (median 14.28 vs 21.35 U/mL, *P* ═ 0.024) (normal value < 37 U/mL) than those with GBADC before matching. Patients in the GBNEC group showed more aggressive tumor behaviors, including more advanced tumor stages (*P* ═ 0.003), lower differentiation (*P* < 0.001), a higher rate of PNI (55.6% vs 22.4%, *P* < 0.001), and a higher incidence of LVI (63.90% vs 45.80%, *P* ═ 0.658) (Table 2).

#### After propensity score matching

**Figure S1.**
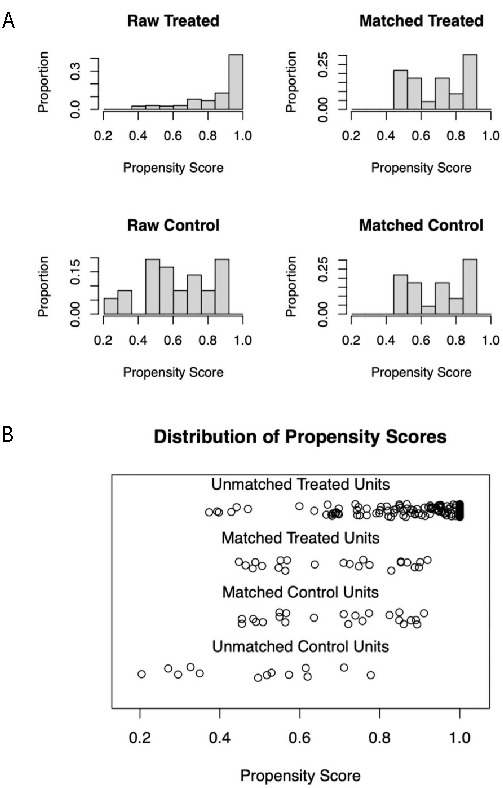
**Plots of distribution of propensity scores.** (A) Propensity score proportion histograms for the unmatched and matched cases and controls; (B) Propensity score jitter plot for the unmatched and matched cases and controls.

After a 1:1 PSM based on seven predefined variables: age, sex, the AJCC stage, resection status, PNI, LVI, and degree of tumor differentiation was performed, the two cohorts were well-matched (Figure S1A and S1B). There was no significant difference in the patients’ baseline characteristics and tumor oncological features (Table 2). Patients with GBADC were associated with higher serum CA 125 levels than those with GBNEC, with a median value of 21.88 U/mL compared to 16.09 U/mL in the GBNEC group (normal value < 35 U/mL) (Table 2).

### Survival prognosis before and after propensity score matching analyses

**Figure 2.**
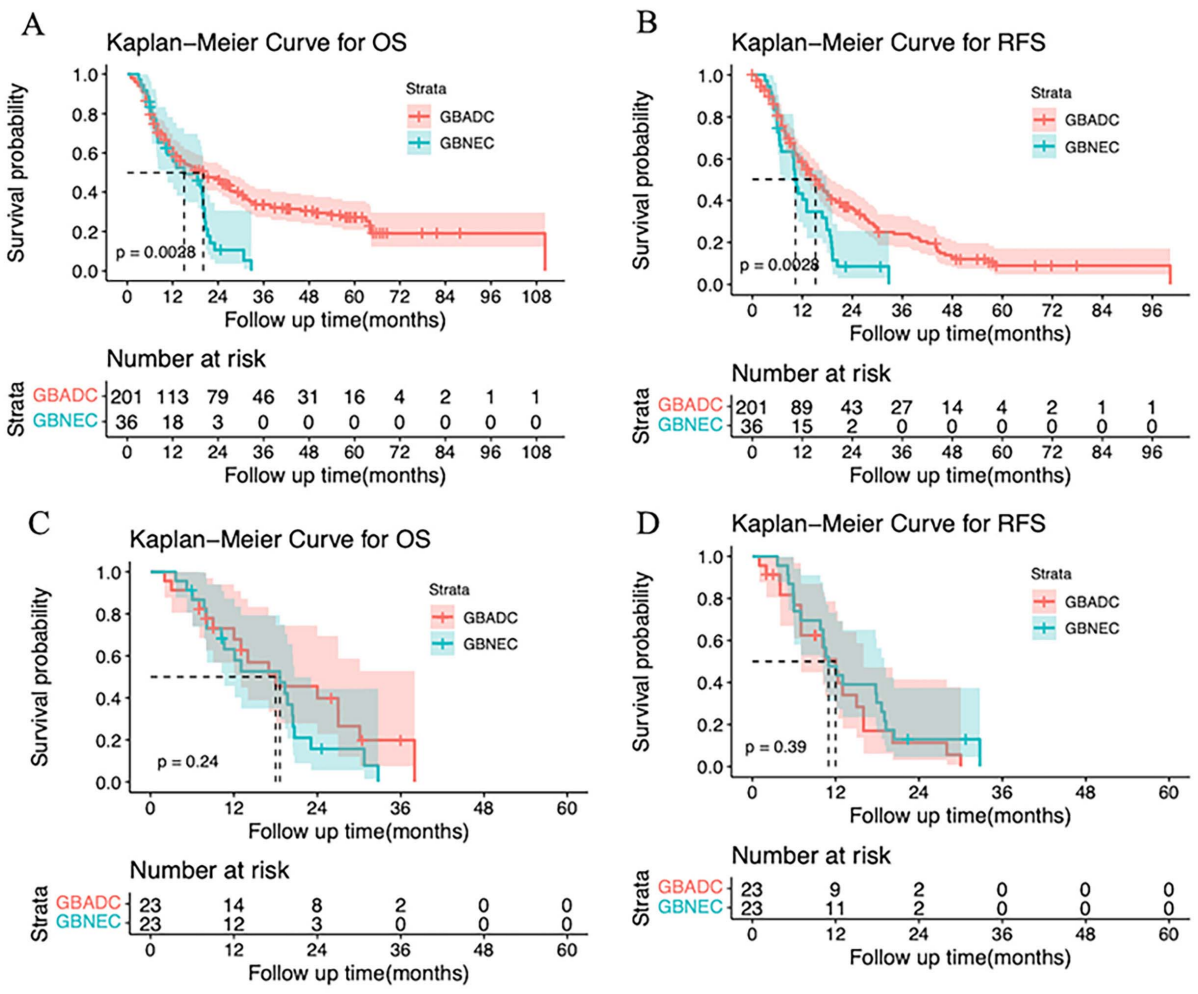
(A and B) Comparison of the OS and RFS between patients with GBNEC and GBADC before PSM; (C and D) Comparison of the OS and RFS between patients with GBNEC and GBADC after PSM. GBNEC: Gallbladder neuroendocrine carcinoma; GBADC: Gallbladder adenocarcinoma; PSM: Propensity score matching; OS: Overall survival; RFS: Recurrence-free survival.

**Figure S2.**
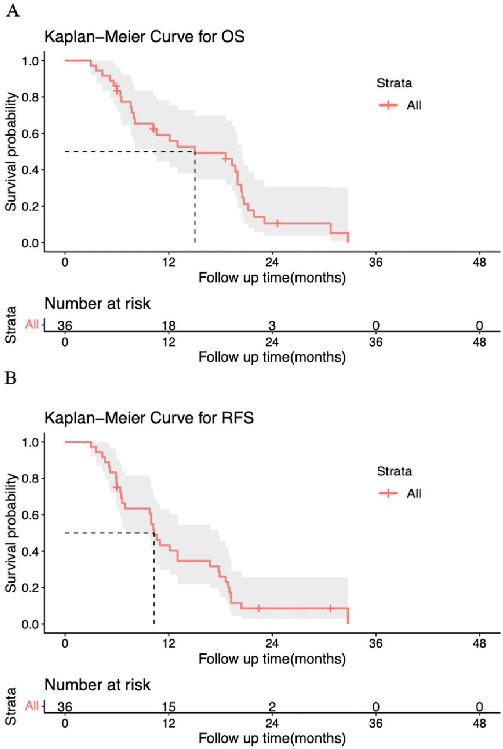
**Plots of (A) OS and (B) RFS for included GBNEC patients.** GBNEC: Gallbladder neuroendocrine carcinoma; OS: Overall survival; RFS: Recurrence-free survival.

In the pre-matching analyses for GBNEC and GBADC, patients with GBNEC had significantly worse OS than those with GBADC with a median OS (mOS) of 15.02 vs 20.11 months, respectively (*P* ═ 0.0028) (Figure 2A). As shown in Figure 2B, patients with GBNEC also had poorer RFS than patients with GBADC, with a median RFS (mRFS) of 10.30 vs 15.17 months, respectively (*P* ═ 0.0028). However, in the matched cohort, the OS for GBNEC was comparable to that for GBADC, with an mOS of 18.6 vs 18.0 months, respectively (*P* ═ 0.24). Similar results were also found in the analyses of mRFS between GBNEC and GBADC (10.98 vs 12.02 months, *P* ═ 0.39) (Figure 2C and 2D). The OS and RFS of the patients with GBNEC are presented in Figure S2. 

**Figure 3.**
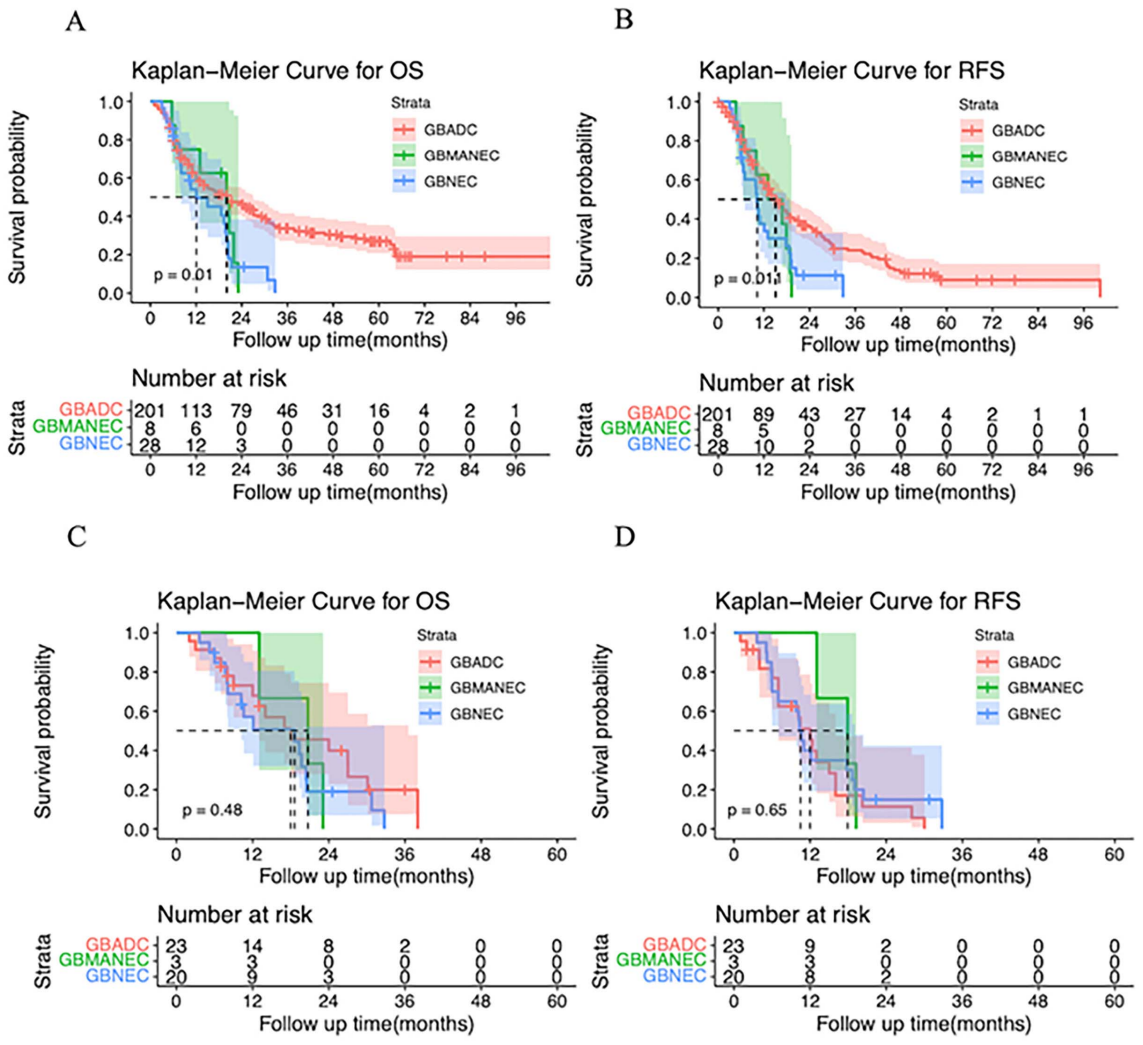
(A and B) Comparison of the OS and RFS between patients with GBNEC, GBMANEC, and GBADC before PSM; (C and D) Comparison of the OS and RFS between patients with GBNEC, GBMANEC, and GBADC after PSM. GBNEC: Gallbladder neuroendocrine carcinoma; GBADC: Gallbladder adenocarcinoma; GBMANEC: Gallbladder mixed adeno-neuroendocrine carcinoma; PSM: Propensity score matching.

Subgroup analyses for different types of GBNEC in the pre-matched cohort found that patients with either GBNEC or GBMANEC had poorer OS (mOS: 18.6 vs 20.7 vs 18.0 months; *P* ═ 0.01) and RFS (mRFS 10.45 vs 17.89 vs 12.0 months; *P* ═ 0.01) compared to patients with GBADC (Figure 3A and 3B). However, after PSM analyses, we found comparable mOS (18.6 vs 20.7 vs 18.0 months, *P* ═ 0.48) and mRFS (10.45 vs 17.89 vs 12.00 months, *P* ═ 0.65) to patients with GBADC (Figure 3C and 3D).

### Prognostic factors in resected GBNEC and GBADC patients

We found that patients with resected GBNEC showed aggressive tumor biological features and worse prognosis. To identify independent prognostic factors for OS and RFS in included patients, the clinicopathological factors were analyzed using univariate and multivariate Cox proportional hazards regression models (Tables 3 and 4).

**Table 3:** Univariate and multivariate Cox regression analyses of predicting overall survival for the included GBNEC and GBADC patients

**Variables**	**Univariate analysis HR (95% CI)**	***P* value**	**Multivariate analysis HR (95% CI)**	***P* value**
Sex (female vs male)	1.16 (0.84 – 1.61)	0.370		
Age (≤60 vs >60 years)	1.00 (0.98 – 1.01)	0.717		
Tumor diagnosis (GBNEC vs GBMANEC vs GBADC)	1.81 (1.20 – 2.72)	**0.004**	0.77 (0.17 – 3.5)	0.731
Tumor stages (I vs II vs III vs IV, AJCC 8th)	4.40 (3.42 – 5.66)	**<0.0001**	2.61 (1.46 – 4.65)	**0.001**
Tumor differentiation (well/moderate vs poor)	0.30 (0.22 – 0.41)	**<0.0001**	0.29 (0.15 – 0.56)	**<0.0001**
LVI (presence vs absence)	8.28 (5.75 – 11.93)	**<0.0001**	9.82 (4.81 – 20.05)	**<0.0001**
PNI (presence vs absence)	3.38 (2.35 – 4.84)	**<0.0001**	0.71 (0.42 – 1.22)	0.217
Jaundice (presence vs absence)	1.31 (0.83 – 2.07)	0.252		
Resection margins (R1 vs R0)	4.43 (3.03 – 6.48)	**<0.0001**	4.55 (1.69 – 12.23)	**0.003**
Gallstone (presence vs absence)	1.24 (0.91 – 1.69)	0.174		
CA 125 group (≥35 vs <35 U/mL)	3.02 (2.18 – 4.19)	**<0.0001**	1.03 (0.62 – 1.71)	0.922
CA 19-9 group (≥37 vs <37 U/mL)	1.87 (1.37 – 2.55)	**<0.0001**	1.09 (0.71 – 1.67)	0.698
Adjuvant radio-chemotherapy (yes vs no)	1.26 (0.80 – 1.90)	0.317		

Bold values indicate statistical significance. CI: Confidence interval; HR: Hazard ratio; GBNEC: Gallbladder neuroendocrine carcinoma; GBADC: Gallbladder adenocarcinoma; PNI: Perineural invasion; LVI: Lymphovascular invasion; GBMANEC: Gallbladder mixed adeno-neuroendocrine carcinoma; AJCC: The American Joint Committee on Cancer; CA 125: Carbohydrate antigen 125; CA 19-9: Cancer antigen 19-9.

**Table 4:** Univariate and multivariate Cox regression analyses of predicting recurrence-free survival for the included GBNEC and GBADC patients

**Variables**	**Univariate analysis HR (95% CI)**	***P* value**	**Multivariate analysis** **HR (95% CI)**	***P* value**
Sex (female vs male)	0.99 (0.98 – 1.01)	0.455		
Age (≤60 vs >60 years)	1.02 (0.74 – 1.4)	0.926		
Tumor diagnosis (GBNEC vs GBMANEC vs GBADC)	1.79 (1.21 – 2.65)	**0.003**	0.63 (0.16 – 2.49)	**0.506**
Tumor stages (I vs II vs III vs IV, AJCC 8th)	4.01 (3.19 – 5.04)	**<0.0001**	7.96 (4.21 – 15.06)	**<0.0001**
Tumor differentiation (well/moderate vs poor)	0.26 (0.18 – 0.37)	**<0.0001**	0.39 (0.22 – 0.69)	**0.001**
LVI (presence vs absence)	7.30 (5.05 – 10.55)	**<0.0001**	3.70 (1.72 – 7.95)	**0.001**
PNI (presence vs absence)	4.46 (3.08 – 6.44)	**<0.0001**	1.05 (0.55 – 1.99)	0.883
Jaundice (presence vs absence)	1.24 (0.78 – 1.96)	0.366		
Resection margins (R1 vs R0)	5.24 (3.52 – 7.81)	**<0.0001**	3.36 (1.03 – 10.94)	**0.044**
Gallstone (presence vs absence)	1.13 (0.83 – 1.55)	0.428		
CA 125 group (≥35 vs <35 U/mL)	2.27(1.61 – 3.20)	**<0.0001**	0.99 (0.55 – 1.79)	0.979
CA 19-9 group (≥ 37 vs <37 U/mL)	1.59 (1.16 – 2.17)	**0.004**	0.98 (0.6 – 1.61)	0.946
Adjuvant radio-chemotherapy (yes vs no)	1.17 (0.77 – 1.78)	0.461		

Bold values indicate statistical significance. CI: Confidence interval; HR: Hazard ratio; GBNEC: Gallbladder neuroendocrine carcinoma; GBMANEC: Gallbladder mixed adeno-neuroendocrine carcinoma; GBADC: Gallbladder adenocarcinoma; PNI: Perineural invasion; LVI: Lymphovascular invasion; CA 19-9: Cancer antigen 19-9; CA 125: Carbohydrate antigen 125; AJCC: The American Joint Committee on Cancer.

The univariate Cox analysis for the enrolled gallbladder cancer patients showed that patients’ tumor diagnosis of GBNEC (hazard ratio [HR] 1.81, *P* ═ 0.004), advanced tumor AJCC stages (HR 4.40, *P* < 0.0001), well/moderate differentiation degree (HR 0.30, *P* < 0.0001), presence of LVI (HR 8.28, *P* < 0.0001) or PNI (HR 3.38, *P* < 0.0001), and positive surgical resection (R1) (HR 4.43, *P* < 0.0001), CA 125 group (≥ 35 vs < 35 U/mL; HR 3.02, *P* < 0.0001), CA 19-9 group (≥ 37 vs < 37 U/mL; HR 1.87, *P* < 0.0001) were significantly associated with patients’ postoperative OS. However, in the multivariate analysis, tumor diagnosis (GBNEC vs GBMANEC vs GBADC; HR 0.77, *P* ═ 0.731), CA 125 (HR 1.03, *P* ═ 0.922), or CA 19-9 (HR 1.09, *P* ═ 0.698) serum levels were not an independent risk factor for OS. Unfavorable pathological features including advanced AJCC stages (HR 2.61, *P* ═ 0.001), well/moderate differentiation degree (HR 0.29, *P* < 0.0001), the neoplasm presence of LVI (HR 9.82, *P* < 0.0001), and patients after R1 resection (HR 4.55, *P* ═ 0.003) are the main determinants of patients’ OS (Table 3).

The univariate analysis results showed that only tumor diagnosis of GBNEC (HR 1.79, *P* ═ 0.003), advanced tumor AJCC stages (HR 4.01, *P* < 0.0001), well/moderate differentiation degree (HR 0.26, *P* < 0.0001), presence of LVI (HR 7.30, *P* < 0.0001) or PNI (HR 4.46, *P* < 0.0001), and positive surgical resection (R1) (HR 5.24, *P* < 0.0001), CA 125 group (≥ 35 vs < 35 U/mL; HR 2.27, *P* < 0.0001), CA 19-9 group (≥ 37 vs < 37 U/mL; HR 1.59, *P* ═ 0.004) were associated with RFS. Multivariable analysis showed that unfavorable pathological features, including advanced AJCC stages (HR 7.96, *P* < 0.0001), well/moderate differentiation degree (HR 0.39, *P* ═ 0.001), the neoplasm presence of LVI (HR 3.70, *P* ═ 0.001), and patients after R1 resection (HR 3.36, *P* ═ 0.044) are independent prognostic factors for RFS (Table 4).

## Discussion

In the present study, we found that patients with GBNEC generally had significantly worse prognoses than patients with GBADC. However, there was no statistically significant difference in postoperative OS and RFS between GBNEC and matched poorly differentiated GBADC. Moreover, we noticed that unfavorable pathological features, such as advanced tumor stages, poor differentiation, LVI, and positive surgical margins, are the main factors influencing patients’ prognosis. The tumor diagnosis itself (GBADC or GBNEC) was not identified as an independent risk factor.

Concerning the group of poorly or undifferentiated tumors, the prognosis of patients with GBNEC is poor. However, there is no general agreement on whether the prognosis of GBNEC is worse than that of GBADC [25–28]. A retrospective analysis by Chen et al. [8] included 10 patients with GBNEC and showed a median survival of 3.0 months, along with 1-, 2-, and 3-year cumulative survival rates of 20%, 10%, and 0%, respectively. In contrast, 377 patients with GBADC treated during the same period had a median survival of 6.0 months, with 1-, 2-, 3-, and 5-year survival rates of 38.0%, 31.0%, 30.1%, and 28.4%, respectively. In Yan et al.’s PSM study [9] of 15 patients with GBNECs and 30 patients with GBADCs, survival analysis also showed that patients with GBNECs had a worse OS than those with GBADCs (3-year OS 31.1% vs 63.8%). In our study, the OS and RFS of GBNEC were significantly poorer than those of GBADC before PSM. The result also highlighted the dismal prognosis of NEC originating from the gallbladder, as supported by other literature. However, in the PSM study by Do et al. [11], the authors reported that no significant differences in OS were found between GBNEC and matched GBADC (mOS of 460 and 545 days). In 2015, Yun et al. [27] compared the 5-year OS of 4 patients with GBNECs and 38 patients with GBADC and also found no significant differences between the two groups. Interestingly, our finding is consistent with these reports. After PSM analyses with predefined variables including age, sex, tumor AJCC stages, differentiation, resection margins, PNI, and LVI, we further noticed that OS could be similar in GBNEC and matched poorly differentiated GBADC patients. However, another study by Yan et al. [9] reported that even after PSM analyses, patients with GBNECs had a significantly worse OS than those with GBADCs. In this small retrospective study [9], 5 patients with GBMANEC were included in a group of 15 patients with GBNECs. Patients with NEC originating from the gallbladder have a dismal prognosis; however, studies discussing the prognostic differences between GBADC and GBNEC offer inconsistent results.

What is the main cause of the different outcomes reported in published studies comparing GBADCs and GBNECs? Due to its non-specific clinical manifestations, GBNEC is often difficult to diagnose preoperatively [2, 16, 26, 28]. GBNEC cases are usually diagnosed postoperatively through pathological examination, and most are at advanced stages. In our study, only two GBNEC patients were at AJCC stage II after postoperative diagnosis. In the study by Do et al. [11], all included patients were at stages IIIB and IV (A/B). Although Chen et al. [8] and Yan et al. [9] reported a significantly worse OS for GBNEC compared to GBADC, Chen et al.’s retrospective comparison of 10 GBNEC patients with 377 GBADC patients that revealed most of the included GBNEC patients had advanced tumor stages with mOS of only 3.0 months (only 1 patient at AJCC stage I-III and 9 patients at AJCC stage IV). Similarly, in the study by Yan et al. [9] there were 10 (33.3%) patients in the GBADC group vs 4 (26.7%) in the GBNEC group.

Unlike GBNEC, GBADC can be diagnosed in early TNM stages. For example, some GBADC are incidentally diagnosed after laparoscopic cholecystectomy for benign gallbladder diseases, such as gallstones and gallbladder polyps; these patients usually have early-stage tumors and better OS compared with GBADC patients who have advanced-stage tumors [29]. Thus, in some preliminary comparisons [8, 27] between GBNEC and GBADC, the tumor stages were not comparable between the groups. Patients with GBNEC usually have more advanced stages of tumors, resulting in significantly poorer OS than that of GBADC patients. It seems that the overall higher staging of GBNEC compared to GBADCs may explain the observed poorer prognosis of GBNEC.

In addition to tumor stages, factors like patient demographics, pathological features of the tumor, surgical-related outcomes, postoperative chemotherapy, and other treatments may also be related to inconsistent outcomes when comparing GBNEC and GBADC. Published studies had pointed out that elderly patients with digestive tumors may have higher morbidity and mortality rates after tumor resection. Wang et al. [30] found that older age was independently associated with reduced survival for GBNEC patients after surgical resection. Similarly, the study by Lee and Sung [10] compared the survival prognosis for GBNEC and low-differentiated GBADC and reported worse survival for GBNEC. In their study, the included GBNEC patients were older with a median age of 64 years (range 52–70 years).

The patients’ basic demographics may offer another explanation for the contrary results in the comparison of GBADC and GBNEC prognosis. Due to the aggressive nature of GBNEC, rates of micro-lymphatic and vascular invasion may be higher in the GBNEC group [8, 27]. Lee and Sung [10] reported that the lymphatic invasion rate was positive in 97% of GBNEC patients, compared to 58% in GBADC patients. In most published studies, those factors were related to the poorer prognosis of patients with digestive carcinomas. Unfortunately, those factors were not generally tested in our center. In published studies, those factors were also not considered for comparison [9, 11].

In general, the only curative therapeutic option for GBNEC is surgical resection. In our study, most of the included GBNEC patients underwent curative resections, and the patient survival was significantly longer than in those patients undergoing R1 resections. After multivariate analyses, R1 resection margins were also found as an independent prognostic factor associated with poor survival. However, due to GBNEC’s high malignancy and difficulty of early diagnosis, most patients are diagnosed at late stages, missing the opportunity for surgical treatment. There is also a lack of consensus on the optimal surgical methods for GBNEC, owing to the absence of large-sample research. However, it is certain that ensuring sufficient negative margins is a key factor for a good prognosis for GBNEC. For patients undergoing surgical resection, most studies reported an mOS of less than two years; thus, it is difficult to perform an effective tumor recurrence management as the survival duration after recurrence is relatively short. Moreover, the optimal approach to adjuvant therapy for GBNEC is also unknown. Furthermore, considering that most GBNEC patients are diagnosed at an advanced stage, distant metastases may occur shortly after surgical resection. Accurate recurrence time is difficult to record for these patients, as the survival after tumor recurrence is too short, and many patients are subsequently lost to follow-up.

Why did previous clinical studies, as well as the present study, include relatively small sample sizes of patients with GBNEC when compared with that of GBADC? Unlike gastric or intestinal NEC, it is estimated that only 0.5% of neuroendocrine neoplasms occur in the extrahepatic biliary tract, including the gallbladder [25, 31, 32]. Besides, due to GBNEC’s aggressive biological behaviors and frequent diagnosis at advanced tumor stages, patients may miss the opportunity to undergo radical resection. This could explain why most studies focusing on GBNEC include small sample sizes and involve radical resection. Compared with GBADC, the sample size is an important challenge limiting further research for GBNEC.

This study has several limitations. First, our data was collected from a referral medical center, which may introduce issues such as patient information loss and selection bias. Second, the long duration of follow-up may result in poor compliance and follow-up bias, potentially affecting the timeliness of obtaining specific survival outcomes. Our included cases may have been treated in different hospitals, and the therapeutic approaches, living standards, and understanding of the diseases could also produce certain biases regarding prognosis. Finally, the findings need further research due to the insufficient number of patients.

## Conclusion

In summary, GBNEC is generally diagnosed at advanced clinical stages and often shows unfavorable histological features, including higher rates of LVI and PNI. Patients with GBNEC had similar survival prognoses compared to matched low-differentiated GBADC patients. Complete surgical resection (R0) may affect better clinical outcomes in patients with NEC of the GB. Tumor diagnosis (GBADC or GBNEC) was not identified as an independent risk factor for patient survival. Unfavorable pathological features of the neoplasm are the main determinants of patient prognosis. Due to the rarity of GBNEC cases, the above conclusions still need to be confirmed by large-scale clinical studies.

## Supplemental Data
